# Corticosteroids significantly increase cystatin C levels in the plasma by promoting cystatin C production in rats

**DOI:** 10.1080/0886022X.2019.1638798

**Published:** 2019-07-29

**Authors:** Xiao-Ran Zhu, Ning Ge, Yu Wang, Jian-Long Zhai, Chao Liu

**Affiliations:** a Department of Pharmacy, Hebei General Hospital, Shijiazhuang, China;; b The First Cardiology Division, The First Hospital of Hebei Medical University, Shijiazhuang, China;; c School of Medicine, Regenerative Medicine Institute, National University of Ireland Galway, Galway, Ireland;; d Cardiology Division, Hebei General Hospital, Shijiazhuang, China;; e Cardiovascular Research Center, Hebei Medical University, Shijiazhuang, China

**Keywords:** Cystatin C, dexamethasone, inulin clearance, RU 486

## Abstract

**Background:** Several studies have shown that non-renal factors such as corticosteroids may increase plasma cystatin C levels without affecting kidney function. However, the mechanisms underlying this are unclear. We hypothesized that corticosteroids may increase cystatin C levels in the plasma by promoting its production in tissues. In the present study, we aimed to test our hypothesis in rats by investigating the effect of corticosteroids on cystatin C production in tissues and the glomerular filtration rate (GFR), as measured by the gold standard method (i.e., inulin clearance).

**Results:** Dexamethasone treatment was associated with much higher concentrations of cystatin C in all organ tissue homogenates tested. Dexamethasone increased plasma cystatin C levels in rats, without any decrease in renal inulin clearance. The impact of dexamethasone on plasma and organ tissue cystatin C levels was abolished by RU486, indicating the effect was glucocorticoid receptor-mediated.

**Conclusions:** Our study provides direct evidence that corticosteroids may increase cystatin C levels in the plasma by promoting its production, without any decrease in GFR.

## Introduction

The glomerular filtration rate (GFR) is the key element in establishing a prognosis-predicting model, risk stratification, and drug–dose adjustment tools because impaired renal function adversely affects survival, prognosis, and drug pharmacokinetics [1,2]. Cystatin C is a single, non-glycosylated 13.36-kDa protein, endogenously produced at a constant rate in nucleated cells and filtered by the glomerulus without reabsorption. Over the past decade, cystatin C has been proposed as a potentially useful new endogenous marker for GFR because, compared with creatinine, it is less affected by muscle mass [3]. However, several studies have shown that steroidal agents can increase cystatin C levels in the circulation without impairing GFR [4–9]. It has also been reported that diabetes, thyroid, acute kidney injury, cancer, and heart dysfunction can have a significant effect on blood cystatin C levels [10–12]. However, these studies have several limitations. First, they all estimated GFR using creatinine-based equations instead of the gold standard method, i.e., inulin clearance. Second, only a hypothesis that corticosteroids may increase cystatin C production in tissues was proposed without any certain supporting data. Third, the relationship between corticosteroids and cystatin C is not consistent; two reports did not show any association [13,14]. Hence, we designed this study to determine the effect of corticosteroids on cystatin C levels in the plasma, and to test the hypothesis that corticosteroids can increase cystatin C production in tissues.

## Methods

### Materials

Dexamethasone sodium phosphate (DEX) was kindly provided by CSPC Pharma (Shijiazhuang, China). Mifepristone (RU486) and inulin were purchased from Sigma-Aldrich (St Louis, MO, USA). EDTA and aprotinin was kindly provided by the Beijing North Institute of Biological Technology (Beijing, China). A Rat Cystatin C Immunoassay Elisa Kit (Catalog No. MSCTC0) was purchased from R&D systems (Germany).

### Animal experiments

A total of eighteen male Sprague–Dawley (SD) rats (provided by the Center of Experimental Animals of Hebei Province, China) of body weight in the range 280–320 g were housed in a temperature-controlled environment, exposed to a natural photoperiod (light/dark cycle of 12:12 h), and given *ad libitum* access to food and water. All rats were housed for one week to allow them to adapt to their environment before the study protocols were initiated. All experimental protocols were approved by the Institutional Animal Care and Use Committee of Hebei Medical University (SCXK 2018-004). The rats were managed in accordance with the guidelines for Animal Care and Use of Hebei Medical University.

The SD rats were randomly divided into three groups (*n* = 6/group), as follows: (i) CON: rats received the vehicle (i.e., physiological saline administered intramuscularly) as a control; (ii) DEX: rats received DEX (1 mg/kg, intramuscularly) for 2 days; (iii) DEX + RU486: rats received RU486 (100 mg/kg, subcutaneously) for 2 days. RU486 was administered 1 h prior to DEX to block the glucocorticoid receptors [15,16]. All rats were euthanized by cervical dislocation following isoflurane–nitrous oxide anesthesia. The dosage of DEX was determined according to the conversion of human surface area. Our previous experiments found that the effect of dexamethasone on renal water and sodium excretion was time-dependent and reached a plateau at 48 h. Therefore, we chose to take samples over two consecutive days to eliminate the influence of urination on the experimental results [17].

### Measurement of inulin clearance

Inulin clearance (for GFR evaluation) was determined as recently described [18]. Briefly, following the surgical procedure, a loading dose of inulin (100 mg/kg BW diluted in 0.9% NaCl) was administered via the jugular vein. Subsequently, a constant infusion of inulin (10 mg/kg BW in 0.9% NaCl), at an infusion rate of 0.04 mL/min, was begun and maintained until the end of the experiment. Two urine samples were collected at 30 min intervals using bladder catheterization, while blood samples were obtained at the beginning and end of each experiment. Plasma and urine inulin levels were measured using previously reported methods [18]. The inulin clearance rate was expressed as g/min/kg BW.

### Enzyme-linked immunosorbent (ELISA) assay for cystatin C

Frozen tissues were homogenized, then a BCA Protein Assay Kit (Solarbio, Beijing, China) was used for the quantitative determination of total protein concentration in the supernatants. Plasma and tissue supernatants were diluted to suitable concentrations and measured using an ELISA assay kit (R&D Systems, Minneapolis, MN, USA).

### Statistical analysis

All data are presented as means ± standard error of means (SEM). Statistical analyses were performed using the SPSS statistical package (SPSS version 16.0, Chicago, IL, USA). Kruskal–Wallis test was conducted if data of groups were not normally distributed. If variances were homogenous, *post hoc* Tukey’s test was performed. *p* values of <.05 were considered statistically significant (Supplementary material).

## Results

### Effect of DEX on plasma cystatin C levels and inulin clearance

**Figure 1.**
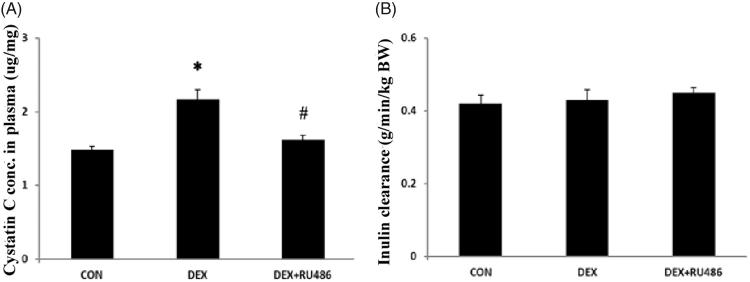
Effects of DEX on plasma cystatin C levels and inulin clearance. (A) Effect of DEX in plasma cystatin C; *p < .05 compared with control; ^#^*p* < .05 compared with DEX. (B) Effect of DEX on inulin clearance.

Following two days of treatment, rats that received DEX had much higher cystatin C levels in their plasma compared with those treated with vehicle (DEX 2.17 ± 0.14 vs CON 1.48 ± 0.04 μg/ml, *p* < .05, Figure 1(A)). The effect of DEX on cystatin C levels was totally abolished by the glucocorticoid receptor (GR) antagonist RU486 (DEX 2.17 ± 0.14 vs DEX + RU486 1.62 ± 0.06 μg/mg, *p* < .05, Figure 1(A)). However, there were no differences between the three groups in their renal inulin clearance (Figure 1(B)).

### Effect of DEX on the production of cystatin C in tissues

**Figure 2.**
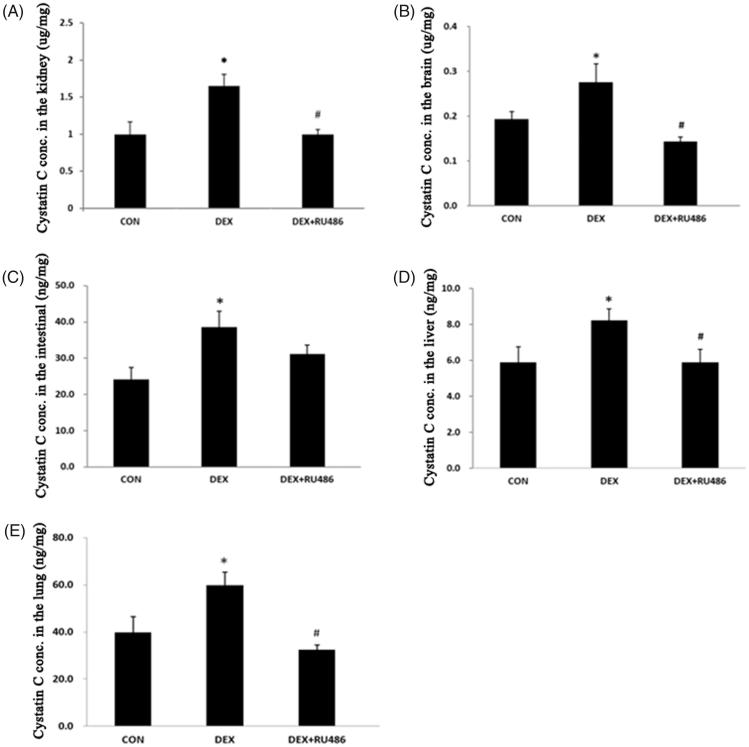
Effect of DEX on the production of cystatin C in tissues. (A). Effect of DEX on cystatin C concentration in the kidneys; (B) Effect of DEX on cystatin C concentration in the brain; (C) Effect of DEX on cystatin C concentration in the intestinal; (D) Effect of DEX on cystatin C concentration in the liver; (E) Effect of DEX on cystatin C concentration in the lung. *p < .05 compared with control; ^#^*p* < .05 compared with DEX.

To test the effect of DEX on the production of cystatin C in tissues, we collected five organ tissue homogenates, from the kidneys, brain, intestines, liver, and lungs. Cystatin C levels were consistently higher in DEX-treated rats compared with those treated with the control (*p*<.05). Consistent with the findings for cystatin C levels in plasma, the effect of DEX on cystatin C levels in the tissue homogenates was also abolished by RU486 (Figure 2(A–E)).

## Discussion

In the present study, we found that the corticosteroid dexamethasone significantly increased cystatin C levels in the plasma of rats and also in tissues, including the kidneys, brain, intestines, liver, and lungs. However, dexamethasone had no impact on GFR as determined by inulin clearance.

Over the past few decades, many methods have been developed to assess GFR; most of these are creatinine-based equations [1,2]. The most commonly used equations are the Cockcroft–Gault equation, the Modification of Diet in Renal Disease (MDRD) Study equation, and the Chronic Kidney Disease Epidemiology Collaboration (CKD-EPI) equation. However, creatinine concentration is dependent on various factors, including muscle mass, age, sex, ethnicity, and food [3,19]. Thus, many correction factors have to be applied when using creatinine-based equations. Nevertheless, the estimated GFR calculated using the above equations is sometimes misleading due to variations in dietary intake (e.g., a vegetarian diet or creatine supplements) or other physiological or clinical conditions. Unlike creatinine-based equations, cystatin C-based estimates for GFR are believed to be less influenced by muscle mass or diet than creatinine-based estimates [1,2]. Cystatin C is ubiquitously expressed at moderate levels in a variety of human biologic fluids; these levels are unaffected by age, sex, or lean tissue mass. Cystatin C is endogenously generated at a stable rate, freely filtered in the glomerulus without reabsorption and secretion in the renal tubule, and not extra-renally eliminated. Therefore, cystatin C has been proposed as an endogenous GFR marker and it has been begun to be used instead of serum creatinine due to its greater reliability and accuracy [20]. However, several studies have shown that non-renal factors can affect serum cystatin C levels and that these should be taken into account when interpreting cystatin C levels [4–9,21–24]. The most important of these non-renal factors is glucocorticoid treatment. In 1995, Bjarnadottir et al first reported that dexamethasone caused a significant dose-dependent increase in cystatin C secretion by cultivated HeLa cells [25]. They also found that glucocorticoid-induced increases in the secretion of cystatin C were due to a promoter-mediated increase in transcription of the cystatin C gene. In 2001, Risch et al first reported that glucocorticoid treatment in patients who had received a renal transplant was associated with elevated serum cystatin C levels after adjustment with creatinine clearance [4]. Subsequently, several reports reiterated the above findings, that glucocorticoid treatment is associated with an elevation in cystatin C after adjustment with creatinine-based eGFR in various diseased populations [5–9]. In 2013, Yamawaki et al found that DEX enhanced the extracellular secretion of cystatin C in esophageal cancer cells [26], suggesting that DEX treatment may be one of the reasons for the increased serum cystatin C concentration seen in patients with cancer during chemotherapy [11,27]. However, there are several limitations when interpreting these findings. First, the authors only mentioned the hypothesis that glucocorticoids may promote cystatin C production in tissues without providing any supporting data. Additionally, all eGFRs calculated during the studies were based upon creatinine-based equations, and not obtained using the gold standard method, i.e., inulin clearance. Additionally, some researchers have questioned the relationship between cystatin C levels and glucocorticoid treatment [13,14]. Our study provides direct evidence that dexamethasone can increase cystatin C production in tissues, resulting in an increase of cystatin C levels in the plasma, without any decrease in GFR. These effects induced by dexamethasone were abolished by the glucocorticoid receptor inhibitor RU486, indicating that the effects possibility described are glucocorticoid-receptor-mediated.

In addition to glucocorticoid treatment, many other non-renal factors have been reported as having an impact on serum cystatin C levels [21–24]. These non-renal factors include proteinuria, diabetes, inflammation, and body mass index [28,29]. It has been reported that hormones, including growth hormone and thyroid hormones, can increase cystatin C levels in the circulation [22,30], while increased cystatin C levels have also been found in patients with malignant diseases [24,31].

## Conclusion

In conclusion, the data from our study showed that corticosteroids can promote the production of cystatin C in various body tissues, resulting in elevated plasma cystatin C levels, without any negative affect on GFR.

### The implications of our findings

Many physiological processes in addition to GFR, such as generation, tubular reabsorption or secretion, and extra-renal elimination can affect the levels of endogenous GFR markers. Measuring changes in the urinary excretion of endogenous markers can help to assess these physiological processes. For example, urinary creatinine excretion can be measured to help interpret unexpected values for creatinine-based eGFR. Unlike creatinine, the absence of urinary excretion of cystatin C makes it impossible to interpret GFR estimates based upon cystatin C. Therefore, our data suggest that cystatin C levels should be interpreted together with the knowledge of several non-renal factors, such as obesity, inflammation, diabetes, proteinuria, or drugs that may impact plasma cystatin C levels. More importantly, clinicians should incorporate serum creatinine levels or serum creatinine-based eGFR results to assist in the interpretation of serum cystatin C and cystatin-C-based eGFR, especially when unexpected eGFR values are encountered.

## Supplementary Material

Supplemental Material

## Data Availability

The datasets used and/or analyzed during the current study available from the corresponding author on reasonable request.

## Abbreviations

Conc.: concentration
eGFR: estimated GFR
ELISA: enzyme-linked immunosorbent assay
DEX: dexamethasone sodium phosphate
GFR: glomerular filtration rate
RU486: Mifepristone
SD rat: Sprague–Dawley rats

## References

[1] Stevens LA, Coresh J, Greene T, et al Assessing kidney function-measured and estimated glomerular filtration rate. N Engl J Med. 2006;354:2473–2483. 16760447 10.1056/NEJMra054415

[2] Vart P, Grams ME Measuring and assessing kidney function. Semin Nephrol. 2016;36:262–272. 27475657 10.1016/j.semnephrol.2016.05.003

[3] Shlipak MG, Matsushita K, Arnlov J, et al. Cystatin C versus creatinine in determining risk based on kidney function. N Engl J Med. 2013;369:932–943. 24004120 10.1056/NEJMoa1214234 PMC3993094

[4] Risch L, Herklotz R, Blumberg A, et al. Effects of glucocorticoid immunosuppression on serum cystatin C concentrations in renal transplant patients. Clin Chem. 2001;47:2055–2059. 11673383

[5] Bokenkamp A, van Wijk JA, Lentze MJ, et al. Effect of corticosteroid therapy on serum cystatin C and beta2-microglobulin concentrations. Clin Chem. 2002;48:1123–1126. 12089191

[6] Risch L, Huber AR Glucocorticoids and increased serum cystatin C concentrations. Clin Chim Acta. 2002;320:133–134. 11983211 10.1016/s0009-8981(02)00044-x

[7] Abbink FC, Laarman CA, Braam KI, et al. Beta-trace protein is not superior to cystatin C for the estimation of GFR in patients receiving corticosteroids. Clin Biochem. 2008;41:299–305. 18082138 10.1016/j.clinbiochem.2007.11.012

[8] Bardi E, Dobos E, Kappelmayer J, et al. Differential effect of corticosteroids on serum cystatin C in thrombocytopenic purpura and leukemia. Pathol Oncol Res. 2010;16:453–456. 20084479 10.1007/s12253-009-9243-0

[9] Zhai JL, Ge N, Zhen Y, et al. Corticosteroids significantly increase serum cystatin C concentration without affecting renal function in symptomatic heart failure. Clin Lab. 2016;62:203–207. 27012051 10.7754/clin.lab.2015.150701

[10] Al Musaimi O, Abu-Nawwas AH, Al Shaer D, et al. Influence of age, gender, smoking, diabetes, thyroid and cardiac dysfunctions on cystatin C biomarker. Semergen. 2019;45:44–51. 30509849 10.1016/j.semerg.2018.07.005

[11] Jones M, Denieffe S, Griffin C, et al. Evaluation of cystatin C in malignancy and comparability of estimates of GFR in oncology patients. Pract Lab Med. 2017;8:95–104. 28856234 10.1016/j.plabm.2017.05.005 PMC5575377

[12] Yang T, Sun S, Lin L, et al. Predictive factors upon discontinuation of renal replacement therapy for long-term chronic dialysis and death in acute kidney injury patients. Artif Org. 2017;41:1127–1134. 10.1111/aor.12927 28544060

[13] Foster J, Reisman W, Lepage N, et al. Influence of commonly used drugs on the accuracy of cystatin C-derived glomerular filtration rate. Pediatr Nephrol. 2006;21:235–238. 16240157 10.1007/s00467-005-2075-6

[14] Silva MV, Moscoso Solorzano G, Nishida SK, et al. Are serum cystatin C levels influenced by steroid doses in lupus nephritis patients? J Bras Nefrol. 2011;33:306–312. 22042347 10.1590/s0101-28002011000300006

[15] Simola N, Paci E, Serra M, et al. Modulation of rat 50-kHz ultrasonic vocalizations by glucocorticoid signaling: possible relevance to reward and motivation. Int J Neuropsychopharmacol. 2018;21:73–83. 29182715 10.1093/ijnp/pyx106 PMC5795343

[16] Liu C, Chen Y, Kang Y, et al. Glucocorticoids improve renal responsiveness to atrial natriuretic peptide by up-regulating natriuretic peptide receptor-A expression in the renal inner medullary collecting duct in decompensated heart failure. J Pharmacol Exp Ther. 2011;339:203–209. 21737535 10.1124/jpet.111.184796

[17] Liu C, Ge N, Zhai JL, et al. Dexamethasone-induced diuresis is associated with inhibition of the renin–angiotensin–aldosterone system in rats. Kaohsiung J Med Sci. 2016;32:614–619. 27914612 10.1016/j.kjms.2016.09.007 PMC12977107

[18] Aguirre JA, Ibarra FR, Barontini M, et al. Effect of glucocorticoids on renal dopamine production. Eur J Pharmacol. 1999;370:271–278. 10334502 10.1016/s0014-2999(99)00121-1

[19] Oterdoom LH, Gansevoort RT, Schouten JP, et al. Urinary creatinine excretion, an indirect measure of muscle mass, is an independent predictor of cardiovascular disease and mortality in the general population. Atherosclerosis. 2009;207:534–540. 19535078 10.1016/j.atherosclerosis.2009.05.010

[20] Bostan Gayret Ö, Taşdemir M, Erol M, et al. Are there any new reliable markers to detect renal injury in obese children? Ren Fail. 2018;40:416–422. 30035656 10.1080/0886022X.2018.1489284 PMC6060377

[21] Kotajima N, Yanagawa Y, Aoki T, et al. Influence of thyroid hormones and transforming growth factor-beta1 on cystatin C concentrations. J Int Med Res. 2010;38:1365–1373. 20926009 10.1177/147323001003800418

[22] Stevens LA, Schmid CH, Greene T, et al. Factors other than glomerular filtration rate affect serum cystatin C levels. Kidney Int. 2009;75:652–660. 19119287 10.1038/ki.2008.638 PMC4557800

[23] Demirtaş S, Akan Ö, Can M, et al. Cystatin C can be affected by nonrenal factors: a preliminary study on leukemia. Clin Biochem. 2006;39:115–118. 16337174 10.1016/j.clinbiochem.2005.10.009

[24] Knight EL, Verhave JC, Spiegelman D, et al. Factors influencing serum cystatin C levels other than renal function and the impact on renal function measurement. Kidney Int. 2004;65:1416–1421. 15086483 10.1111/j.1523-1755.2004.00517.x

[25] Bjarnadottir M, Grubb A, Olafsson I Promoter-mediated, dexamethasone-induced increase in cystatin C production by HeLa cells. Scand J Clin Lab Invest. 1995;55:617–623. 8633186 10.3109/00365519509110261

[26] Yamawaki C, Takahashi M, Takara K, et al. Effect of dexamethasone on extracellular secretion of cystatin C in cancer cell lines. Biomed Rep. 2013;1:115–118. 24648905 10.3892/br.2012.21 PMC3956473

[27] Kume M, Yasui H, Yoshikawa Y, et al. Transient elevation of serum cystatin C concentrations during perioperative cisplatin-based chemotherapy in esophageal cancer patients. Cancer Chemother Pharmacol. 2012;69:1537–1544. 22437652 10.1007/s00280-012-1860-8

[28] Čabarkapa V, Ilinčić B, Đerić M, et al. Cystatin C, vascular biomarkers and measured glomerular filtration rate in patients with unresponsive hypertensive phenotype: a pilot study. Ren Fail. 2017;39:203–210. 27876431 10.1080/0886022X.2016.1256316 PMC6014334

[29] Deng Y, Wang L, Hou Y, et al. The influence of glycemic status on the performance of cystatin C for acute kidney injury detection in the critically ill. Ren Fail. 2019;41:139–149. 30942122 10.1080/0886022X.2019.1586722 PMC6450510

[30] Sze L, Bernays RL, Zwimpfer C, et al. Impact of growth hormone on cystatin C. Nephron Extra. 2013;3:118–124. 24348508 10.1159/000356464 PMC3861865

[31] Nakai K, Kikuchi M, Fujimoto K, et al. Serum levels of cystatin C in patients with malignancy. Clin Exp Nephrol. 2008;12:132–139. 18317874 10.1007/s10157-008-0043-8

