# Supplementary material for: Corticosteroids significantly increase cystatin C levels in the plasma by promoting cystatin C production in rats
Source: Ren Fail. 2019 Jul 29;41(1):698–703. doi: 10.1080/0886022X.2019.1638798 (PMC6711078; doi:10.1080/0886022X.2019.1638798)
Supplement: Supplemental Material [file IRNF_A_1638798_SM3831.pdf]

Statistical analysis

We used Shapiro-Wilk test to judge the normality of distribution, and then conducted the homogeneity test of variance. For the samples with normal distribution and homogeneity of variance, one-way ANOVA was performed to compare the statistical differences between DEX and CON, RU486+DEX and DEX. For the samples with skewed distributions and unequal variance, we used three different statistical methods to test the difference between the groups. The list is as follows.

Table 1. The effect of dexamethasone on cystatin C in various tissues between groups.

	Test for normality	Test of homogeneity of variance	One-way ANOVA	Tamhane's T2	Dunnett's T3	Kruskal-Wallis test
Plasma	√	×	×	$P < 0.05$	$P < 0.05$	$P < 0.05$
Kidney	√	×	×	$P < 0.05$	$P < 0.05$	$P < 0.05$
Brain	×	√	×	$P < 0.05$	$P < 0.05$	$P < 0.05$
Intestine	√	√	√ $P < 0.05$	$P > 0.05$	$P > 0.05$	$P > 0.05$
Liver	√	√	√ $P < 0.05$	$P > 0.05$	$P > 0.05$	$P > 0.05$
Lung	√	√	√ $P < 0.05$	$P < 0.05$	$P < 0.05$	$P < 0.05$
